# The impact of misinformation on the COVID-19 pandemic

**DOI:** 10.3934/publichealth.2022018

**Published:** 2022-01-12

**Authors:** Maria Mercedes Ferreira Caceres, Juan Pablo Sosa, Jannel A Lawrence, Cristina Sestacovschi, Atiyah Tidd-Johnson, Muhammad Haseeb UI Rasool, Vinay Kumar Gadamidi, Saleha Ozair, Krunal Pandav, Claudia Cuevas-Lou, Matthew Parrish, Ivan Rodriguez, Javier Perez Fernandez

**Affiliations:** 1 Division of Research & Academic Affairs, Larkin Community Hospital, South Miami, Florida, USA; 2 Department of Medicine, American University of Antigua, Coolidge, Antigua; 3 Family Medicine, Larkin Community Hospital Palm Springs Campus, Hialeah, Florida, USA; 4 Family Medicine, Larkin Community Hospital South Campus, Miami, Florida, USA; 5 Pulmonary Disease and Critical Care Medicine, Larkin Community Hospital Palm Springs Campus, Hilaeah, Florida, USA

**Keywords:** COVID-19, healthcare, misinformation, public health, social media

## Abstract

Since the inception of the current pandemic, COVID-19 related misinformation has played a role in defaulting control of the situation. It has become evident that the internet, social media, and other communication outlets with readily available data have contributed to the dissemination and availability of misleading information. It has perpetuated beliefs that led to vaccine avoidance, mask refusal, and utilization of medications with insignificant scientific data, ultimately contributing to increased morbidity. Undoubtedly, misinformation has become a challenge and a burden to individual health, public health, and governments globally. Our review article aims at providing an overview and summary regarding the role of media, other information outlets, and their impact on the pandemic. The goal of this article is to increase awareness of the negative impact of misinformation on the pandemic. In addition, we discuss a few recommendations that could aid in decreasing this burden, as preventing the conception and dissemination of misinformation is essential.

## 1. Introduction

The earliest cases of COVID-19 were reported in Wuhan, China, in December 2019 [1],[2]. Due to its rapid spread worldwide, the World Health Organization (WHO) declared a pandemic [3]–[5]. As of December 20, 2021, there had been 275,007,350 confirmed cases, 5,370,192 reported deaths, and 246,674,846 recovered individuals across the world [6].

Information is the “factual data” that entails the knowledge that flows through society and guides individuals in their choices, actions, efforts, or lack thereof [7]. We obtain and refer to sources of information from numerous entities, including but not limited to television, radio, newspapers, magazines, journals, advertisements, the internet, experts, friends, and the ever-expanding realm of social media. Albeit advantageous when valid, what are the consequences of false, biased, misleading, and erroneous information? Notably, it is imperative to consider that rumors, myths, and opinions also serve as information. As we investigate the current global pandemic, we can perceive first-hand the detrimental effects that its contrary misinformation creates.

Unfortunately, misinformation has a further burden on controlling the situation [8]. It could lead to rumors, stigma, discrimination, false theories and shapes beliefs and attitudes, ultimately creating confusion that curtails the advancement of individual health and public health during crises [9],[10]. Some examples include association of face masks and CO_2_ toxicity, conspiracy theories related to Bill Gates and discussions related to the various non-approved drugs for COVID-19 [11]. To add, health misinformation negatively affects individuals' decisions, leading to poor outcomes in physical health, mental health, and continued viral spread [12].

One of the most well-known examples of misinformation is the potential link of the MMR vaccine with autism and gastroenteritis in children [13]. Later retracted from The Lancet, the claims stemmed from a 1998 published paper, creating confusion and ultimately dire health consequences as parents grounded on misinformation from published findings declined the vaccination of their child [13]. Data collected estimated that, in 2019, there were 100,000 cases of measles in Europe [14]. In Romania, during 2016 to 2018, there were 12,918 cases [14]. During this period, New York City recorded 649 measles cases between September 2018 and July 2019, with 85% of those affected, unvaccinated [15]. In an article published in 2016, it was seen that out of 574 cases of measles among unvaccinated subjects, 70.6% were unvaccinated, further highlighting vaccine hesitancy among a misinformed public [16]. A systematic review found that parents who refused vaccination or were hesitant not only withheld concerns regarding the effectiveness and safety of the MMR vaccine but did not trust the experts, healthcare workers, government or their intentions. Further, they obtained information from the media and lay public or perspectives [17]. Apart from inaccurate data, misinformation also results when a society loses trust in health care due to previous experiences of health inequalities based on race, socioeconomic status, and education [8].

Not only has curtailing the spread of COVID-19 proved challenging for global health, but so too does the confusion and misinformation that accompanies and further worsens health outcomes and disease burden [12]. From its inception to the present, misinformation has governed an individual's choice to increase handwashing, adhere to and maintain social distancing, comply with mask mandates, and initiate vaccination [8].

Several myths have also become common hearsay such as vaccines negatively affecting fertility in women and vaccines altering the genetic makeup of recipients [18]. Misleading information also target beliefs for management and treatment — the consumption of alcohol, cow excrements, cow urine, colloidal silver, teas, and essential oils have all been associated with curing COVID-19 infection, but all examples of misinformation, strong scientifically proven evidence to support the effectivity of any is yet to be seen [19].

As the generation of information is theoretically free, the scope of misinformation is numerous and limitless. Consequently, medical and governing entities must assert that accurate, evidence-based information dominates science and public health to control and limit the dissemination of falsities of misinformation. As coined by the WHO, the pandemic has also spawned an “infodemic”, the plethora of information created during a health crisis, which is misleading and untrue. Accompanying its health woes is the counter-productive beliefs and behaviors that worsen, lengthen, and further complicate the health predicament [20].

Thereby, we present an article on misinformation and COVID-19. This literature review aims at providing an overview and summary regarding the role of media, other information outlets, and their impact on the pandemic. The goal of this article is to increase awareness of the negative impact of misinformation on the pandemic. In addition, we discuss a few recommendations that could aid in decreasing this burden, as preventing the conception and dissemination of misinformation is essential. We also discuss how society could gain public trust in healthcare through honest, clear, reliable scientific knowledge in order to advance public health policies and help control the current global public health emergency.

## 2. Methods

We perform a thorough literature search on databases such as Google, Pubmed, and Google Scholar with the last search date being 10th of November 2021. Articles addressing the impact and role of misinformation during the COVID-19 pandemic were included. We aim to determine the role of this burden and create awareness of its negative impact during pandemic events. The key descriptors used for the search purposes were COVID-19, Coronavirus, SARS-CoV-2, communication, healthcare, misinformation, and social media. In addition to the database searching, a few articles were located using the references used at the end of each article. The articles chosen were most relevant to our goal and purpose of this literature review.

## 3. Misinformation sources, risks and preventive measures

### 3.1. Vaccine misinformation and its risk

Since declaring COVID-19 a pandemic by the WHO, herd immunity by vaccination was speculated to be the ultimate remedy to overcome the situation. However, the extent of vaccination for herd immunity for COVID-19 remains to be known [21]. Presently, 4.76 billion doses of various COVID-19 vaccinations have been administered [22]. Worldwide, 23.7% of the global population is fully vaccinated, with 31.7% receiving their first vaccine dose [23].

Vaccine fear is a predominant public health issue, and a similar trend of misconceptions was observed in relation to the COVID-19 vaccine upon its introduction [24]. Concerns related to the COVID-19 vaccine reported to the Centers for Disease Control and Prevention (CDC) included not only its effect on fertility in women and the ability to alter the human genome but also beliefs regarding a global effort to decrease world population and skepticality regarding its emergency authorization granted by US Food and Drug Administration (FDA) [25].

Numerous other examples of vaccine hesitancy exist. A scoping review of literature from 2000–2020 analyzed 100 articles about vaccine hesitancy prior to the COVID-19 pandemic, in which it was identified gaps prior to the pandemic era in 5 areas such as disciplinary focus; specific vaccine, condition, disease focus, geographical focus; stakeholders and implications; research methodology and it demonstrated that 35 articles were related to vaccine hesitancy of measles, mumps, rubella (MMR), and human papillomavirus (HPV) [26]. Recently, increasing hesitancy regarding the COVID-19 vaccine in health care workers who were not involved in the direct care of the COVID-19 patients was reported [27]. A systematic review encasing 126 peer-reviewed articles and surveys demonstrated decreasing vaccine acceptability from >70% in March 2020 to <50% in October 2020 [28]. Another noticeable trend was that even persons receptive to vaccination preferred to observe the vaccine's response in other people before getting it themselves. Additionally, it was observed that people were more receptive to the vaccine when it was recommended by their primary care provider or based on a self-perception of being a high-risk candidate for COVID-19 infection [29]. In term, this increasing hesitancy has impacted the delivery of health care services globally.

The tendency of an increased risk of vaccine-preventable diseases is seen worldwide due to COVID-19 [30]. Also, an increase in advanced lung cancer cases among patients due to hesitancy to approach health care facilities is seen. In addition, there is a deterioration in health standards among already established patients [31]. Similar trends were reported related to decreasing standard of care for patients suffering from optical diseases, breast cancer, and hearing challenges [32]–[34]. COVID-19 vaccine hesitancy is also reflected in the decreasing frequency of routine childhood immunization [35],[36]. Consequent to these decreasing trends, underdeveloped countries with low vaccination rates are experiencing the 4th wave of COVID-19 cases surge.

### 3.2. Social media influence

Various sources on the internet, including social media, became an outlet for spreading false and inaccurate information related to the pandemic. These include theories about its origin, transmission, prevention, and the effectiveness of vaccines, all of which are not supported by evidence-based findings [37].

**Figure 1.**
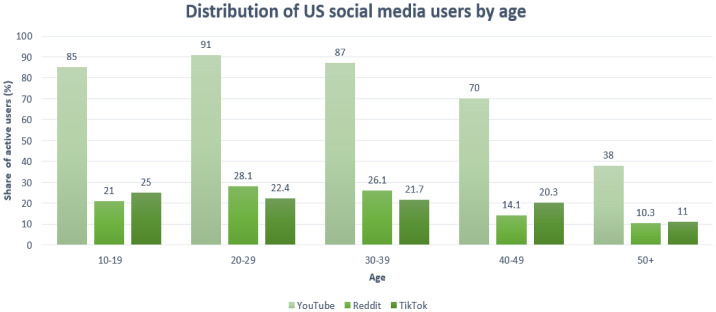
Distribution of US social media users by age.

**Figure 2.**
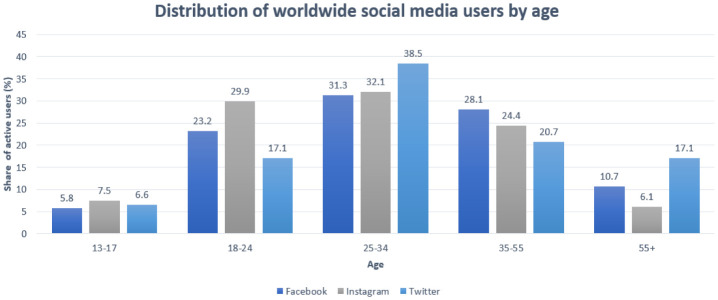
The distribution of the Facebook, Instagram, and Twitter users by age.

With an incidence of 53.6% of the world's population using social media platforms, it has become one of the major outlets for the rapid dissemination of information and thus has significant impacts on misinformation [38]. Facebook roughly has 2.89 billion monthly active users, followed by YouTube with over 2 billion users [38]. At the beginning of 2021, 3.51 billion people were using at least one of the following applications: Facebook, WhatsApp, Instagram, or Messenger, as per US Facebook, Inc [39]. The next in the top are TikTok with over 1 billion users [40], Twitter with around 186 million users [41], and Reddit with about 52 million daily active users [42]. The distribution of YouTube, Reddit, and TikTok by age in the US is reported in Figure 1. The distribution of YouTube, Reddit, and TikTok by age in the US is reported in Figure 1. The distribution of the Facebook, Instagram, and Twitter users worldwide by age is reported in Figure 2.

Predicated on the graph above, it can be stated that people between the ages of 18 to 55 are the most active social media users worldwide. Some of them use more than one application [39]. Therefore, accurate data on the exposure to specific information is difficult to appraise [39].

From Figure 1, it can be concluded that the most popular platform in the US is undoubtedly YouTube, but overall, the usage is almost uniformly distributed in the age range between 10–40 years [39]. Combining these numbers makes it clear that the most susceptible groups to virtual information are teenagers and young adults, followed by a slightly lower percentage by middle-aged adults. That unequivocally leads to a faster spread of information around social circles and a bigger wave of response to news and latest facts, especially during the pandemic.

During the pandemic, Twitter, one of the most popular social media platforms, was heavily used by users to voice their feelings about the disease's spread, share supposed preventions and cures, hypothesize about the disease origin, and how governments should react appropriately, and thus it served as a channel for the spread of both false, partially false, and true claims. In a study to investigate COVID-19 misinformation on Twitter, over 92 expert fact-checking groups analyzed data consisting of false or partially false tweets [43]. Out of 1500 tweets utilized for the study, 1274 were false and 226 partially false assertions [43]. Data also shows that the transmission of misinformation on Twitter was strongly inﬂuenced by verified twitter handles from organizations or celebrities, and incorrect statements spread faster than partially false claims [43].

The term “mainstream media” refers to traditional newspapers, television, and other news sources that most people are familiar with and believe to be trustworthy [44]. These mainstream media outlets have social media accounts on sites like Twitter, which they used to disseminate information during the pandemic. There is mounting evidence that some mainstream media social media accounts either intentionally or inadvertently propagate COVID-19 misinformation. In an analysis into how the AstraZeneca COVID-19 vaccine was discussed by the media on Twitter data shows that popular tweets about AstraZeneca that provoke fear are shared not just by influential activists and conspiracy sites, but also by state-owned social media accounts with the help of bot networks [45]. Exposure to U.S.-based media sites has been linked to COVID-19 misperceptions, and increasing exposure to U.S.-based material on Twitter has been linked to a higher risk of posting misinformation [46].

Social media also had an influence in the spread of information related to vaccines. Vaccines have moved from obscurity to prominence on the front pages of major news publications as a result of COVID-19. Amidst a perceived increase in skepticism, mainstream web media, which is most people's major source of information, has been overwhelmingly favorable regarding vaccines in comparison to news before the pandemic, which was primarily negative [47].

Unquestionably, social media platforms were of great support in helping to maintain communication with family and friends. However, they were aided in reducing boredom, distress, and depression during periods of lockdown. Notably, social media outlets served to disseminate official information to the public and to be able to respond adequately to their questions and concerns. Other significant advantages of social media platforms are the possibility to hold conferences, collaborate with doctors worldwide on research, continue work and education, and create health-oriented projects and applications (apps.) mentioned in this manuscript [48].

Social media corporations took a sequence of measures in collaboration with health organizations and the government to prevent harmful effects discussed below. Besides the “Stop the Spread” “Reporting the Misinformation” app. usage, people are also encouraged to search information via hashtags to make it easier to find the right source. The CDC recommends using #COVID19 when searching for information about the current virus [49].

On May 21, 2020, a new project was launched, called #ShareTheMicNow [50]. It allows famous people to hand over their social media accounts to medical experts to post valid data. One of the first stars to accept this challenge was Julia Roberts, who gave her Instagram account access to Dr. Anthony Fauci, Gwyneth Paltrow, Cheryl Strayed, and Senator Elizabeth Warren [50]. Another example is the collaboration between Vietnamese singer Khac Hun and Vietnam's National Institute. They created a song meant to promote handwashing, and it became viral thanks to a dancer who made a video choreography on TikTok [51].

The United Nations High Commissioner for Refugees (UNHCR) took an important move in dealing with this situation. In March 2020, they posted an article that mentions ten tips that can help assert the validity and impact the information holds [52]. Even though social media can create more distress and even addiction, it is an excellent way to connect the world, and each individual is equally responsible for the information circulating on it.

### 3.3. Importance of trustful sources of information

So how important is it to receive COVID-19 health information from a trustworthy source? If favored over scientific guidelines, misinformation generated by gossip, stigma, and conspiracy theories has serious consequences for public health [9]. Due to misinformation during the first three months of 2020, up to 6000 persons were hospitalized, and 800 were left dead [53]. Obtaining information from an unreliable source has even resulted in death [53].

Since the pandemic's beginning, the internet has played a significant role in disseminating COVID-19 infodemics [54]. Information being reported should be trustworthy as the responses and actions of the public influence the spread and containment of the virus. Misinformation related to COVID-19 has hindered efforts to promote safe practices such as hand washing and social distancing, consequently increasing the spread of the virus [12]. In Brazil, during the first six months of the pandemic, misinformation was mainly focused on the number of cases, deaths, methods of prevention, and treatment [55]. In addition, three main falsified topics circulated on the internet: 1. Field hospitals being empty signified that the virus was not real; 2. Chloroquine and hydroxychloroquine could serve as a cure; and 3. The coffins buried were empty to increase public fear [56]. The falsified statement regarding the coffins caused an increase in the public gathering at burial sites to check for family members, and as a consequence, five people were infected with the virus [56]. By June 2020, 15 people in the US had consumed disinfectant in the misguided idea of preventing the disease [57]. Methanol poisoning claimed the lives of four persons, while three others were left visually impaired [57].

Misinformation has also impacted mental health and can negatively affect recalling past experiences or similar but accurate information heard in the past, a phenomenon known as the misinformation effect [58]. Propagation of misleading information, using “bubble filters”, and an exaggeration of facts caused a wave of stress, anxiety, confusion, and depression amongst the global population [59]. A study demonstrated that social media sites increased anxiety and panic among individuals in Iraqi Kurdistan during the pandemic [60]. Misinformation shared online regarding impending lockdowns during the first several months of the pandemic led to panic buying resulting in a shortage of much-needed supplies [9].

One's first source of information regarding the pandemic should not be from a social media post that can be heavily opinionated or from an unofficial website. The National Institutes of Health (NIH) recommends a list of seven questions a person should ask to ensure that health information online is trustworthy [61]. The main ideas of these questions include the sponsorship of websites, whether or not a health professional wrote the article, clarity of the mission or goal of the website, availability of contact information for the sponsor of the website, last written update to the information, protection of privacy and whether the website promises or provides quick solutions to problems [61].

Receiving information from accurate sources helps empower the public to make important health decisions. Thus, it is crucial to curb the spread of falsified information to prevent further detrimental consequences that place public health at an even greater risk.

### 3.4. Preventive measures against misinformation

Undoubtedly, misinformation leads to uncooperative behavior among the general population. Misleading information spreads in multiple ways, including skepticism and fear induced by medical personnel [62], anti-vaccine campaigns from people seeking financial or political profit [63], rumors, and hoaxes at social gatherings and social media [64].

The primary and perhaps most significant issue regarding misinformation is the lack of relevant information. By February 6, 2020, no quality information was available on the internet about COVID-19 [65]. Educational resources are limited, and health officials/educational campaigns often appraise messages based on what they want to promote, not what is accurate and evidence-based. This raises a certain level of distrust and skepticism in the general population.

To boost their motivation in curbing misinformation, UK Government-WHO collaborative campaigns and US Government-CDC collaborative campaigns created a series of social media infographics and messages to explain the safety of COVID-19 vaccines [53]. Another successful project, “Stop the Spread”, was launched on BBC World television, website, and app. during May and June 2020. Its purpose is to increase the public's awareness of the magnitude of misinformation about COVID-19 and motivate them to verify it and limit the spread, and therefore damage it can cause [66]. Another app, “Reporting Misinformation”, encouraged people to check and report inaccurate information to different social media platforms. It is available in 5 languages and became the second most viewed COVID-19 related page. A subsequent campaign developed in Florida, “Our best shot”, encourages the community to receive vaccination, wear masks, continue hand washing, and maintain social distancing advice. Further, their project included creating a workshop kit equipped with tools and educational materials for community leaders to teach people about vaccination [67].

**Figure 3.**
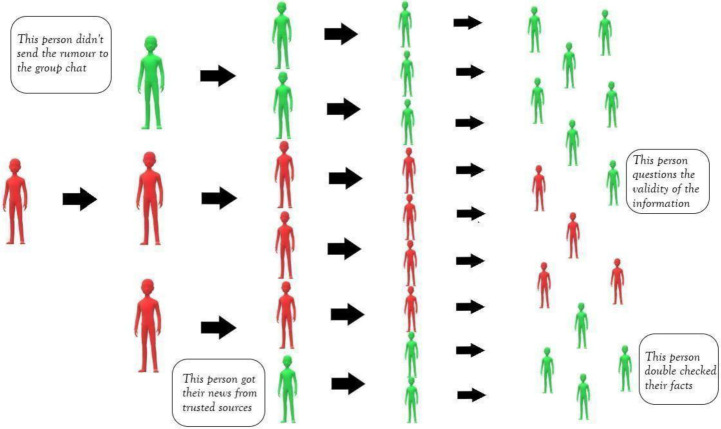
The goal of prebunking.

Prevention is the most effective way to combat misinformation. It uses the same principle as a vaccine: people are exposed to a few erroneous facts, explain how they are misguided, and aver [68]. An interesting strategy called “prebunking”, aims to prevent misinformation in precisely this format. Its method has three main categories: (1) fact-based: rectifying a false statement, (2) logic-based: unraveling the strategies used to misguide and manipulate, and (3) source-based: mentioning sources that spread misinformation. Refer to Figure 3 for more information.

The logic-based approach showed the best results so far [69]. A postdoctoral researcher at the University of Cambridge developed and tested this technique using “Fake News”, a gamified intercession that imitates a social media platform and explains to participants how to detect which news headlines are factual and which are not [70]. When the infodemic struck, Van der Linden and Roozenbeek built a new online game, “Go Viral!”, which aims to prebunk common misinformation surrounding COVID-19.

It showed that just one play could decrease the perceived reliability of fake news by an average of 21%. Participants can display their results on social media platforms and connect to WHO's COVID-19 “Mythbusters” [64]. The game teaches players how conspiracy theories, emotional language, and fake experts can mislead and is undeniable proof that government and health organizations' collaborative efforts can bring sizable results [53].

## 4. Recommendations to decrease the burden

**Figure 4.**
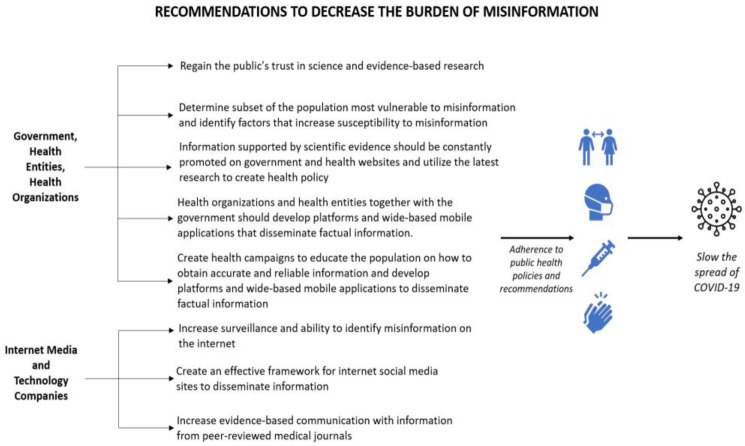
Recommendations to decrease the burden of misinformation.

There needs to be a collaborative approach from the governments of different countries, health organizations, technology companies, news media, and the public to tackle misinformation. We suggest the following recommendations as depicted in Figure 4.

### 4.1. Government, health entities, health organizations

An example of political misinformation during the COVID-19 pandemic happened in Brazil. Their government spread false news on politics, COVID-19 death cases, and its treatment and prevention [55]. During times of crisis, the public's trust in the government increases since reliance falls on public institutions to solve complex problems [71]. Substantial increases in the trust are frequently lost, and by January 2021, worldwide trust in the government had dropped by 8 points, highlighting the difficulties in maintaining high levels of trust over prolonged periods [72]. Throughout the pandemic, misinformation via rumors, conspiracy theories, and stigma led to the public's distrust of recommendations from various international health organizations, governmental public health policies, and vaccine recommendations [9].

Effective implementation of COVID-19 policies requires public compliance, which first requires trust [73]. There is a substantial link between government trust and readiness to engage in prosocial behaviors that aid in the pandemic's control, such as regular handwashing, avoiding crowded settings, and social isolation [73]. There is also a public's distrust of science which should be regained to overcome the COVID-19 pandemic [53]. In increasing public trust, governments should deliver frequent unambiguous messages, appear well organized handling COVID-19 related issues, and offer a greater level of fairness to the public [73]. Also, the government should offer greater accountability and transparency regarding how public health decisions are made in conjunction with various health organizations and entities. Transparency is crucial in times of crisis, and findings show that only 42.3 percent of respondents believe the government communicates the truth regarding COVID-19 most or all of the time, despite 52.7 percent believing the government is making the correct decisions [74].

To tackle misinformation, governments must understand the population most vulnerable and discover factors that increase susceptibility to misinformation [75]. These factors include insufficient access to health information based on evidence and the tendency to have a conspiracy-like mindset [75]. When these vulnerable populations and factors increasing susceptibility are identified, a more strategic approach can be taken when dealing with misinformation in that population subgroup [75].

Governments should work closely with various health entities such as research, university and community hospitals, and health organizations like the WHO to develop effective responses and utilize the latest research to create health policy. In addition, information supported by scientific evidence should continue to be continuously shared on the governmental and health organizations' websites [9].

To overcome the lack of relevant information [65], various health organizations and health entities, together with the government, should develop platforms and wide-based mobile applications (app.) that disseminate factual information. There should also be explicit conferences for medical representatives, informational booklets available in a simple and comprehensive language, and open discussions with the public about all concerning questions. Additionally, health campaigns should be created to educate the population about the different types of information, sources, and how to obtain accurate and reliable information.

### 4.2. Internet media and technology companies

**Figure 5.**
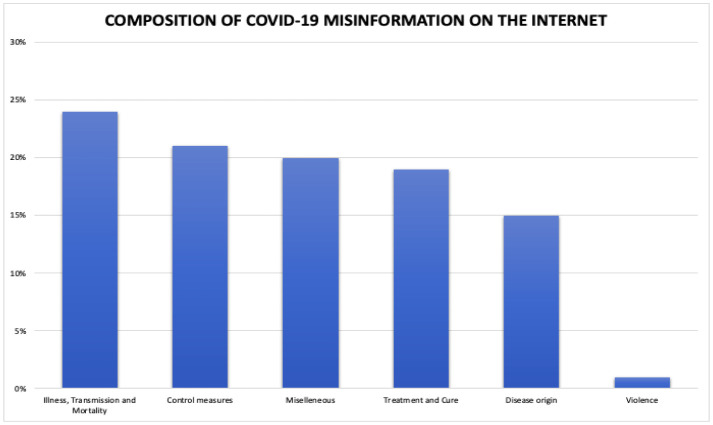
Composition of COVID-19 misinformation on the internet.

Tackling misinformation on the internet firstly involves understanding its composition. Two thousand three hundred eleven online reports of infodemics related to COVID-19 were analyzed and subdivided into the following categories [9] as depicted in Figure 5.

Social media platforms served as a driving force for misinformation related to COVID-19. Thus, one step in dealing with this burden of misinformation on social media is by increasing surveillance of these sites and further enhancing the ability to identify false information on the internet in the form of new software, enhanced algorithms, or increased fact-checkers.

An effective framework for internet media sites to disseminate information should be created to prevent further crises. In creating this framework, the psychological drivers of sharing and accepting false and inaccurate information should be assessed since health topics can lead to anxiety and panic [75]. Media and news websites should increase evidence-based communication with information from peer-reviewed medical journals. Internet media and technology companies can develop apps that disseminate accurate, up-to-date scientific data and government mandates and recommendations based on geography.

## 5. Conclusions

Historically, healthcare misinformation has largely shaped public health behavior, but the impact of this association became ostensive during the COVID-19 pandemic. It brought about a surge of several rumors, hoaxes, and false theories regarding the origin of the virus, its spread, and treatment options which even led to fatalities in some cases. Misinformation regarding the incidence rate, prevalence, and spread of the virus has contributed significantly towards the complacent attitude of people to this crisis. Additionally, the emergency use authorization of various COVID vaccines resurfaced the general public's mistrust in science, which, combined with the rampant spread of falsified information, made vaccine hesitancy a parallel pandemic. As a result, this has shed new light on the importance of having credible sources of healthcare information and continuous monitoring of social media platforms to determine whether accurate information is being relayed to the public. A multifaceted approach is crucial to combat such a massive “infodemic” and requires a collaborative strategy involving local governments and law enforcement bodies, social media companies, community-based organizations, and other vital stakeholders to dispel such myths. This article highlights some of the challenges posed by misinformation in an era of social media, the catastrophic impact of misinformation on fighting public health crises, and the various strategies adopted by countries worldwide to combat it.
